# Analysis and comparison of the wolf microbiome under different environmental factors using three different data of Next Generation Sequencing

**DOI:** 10.1038/s41598-017-11770-4

**Published:** 2017-09-12

**Authors:** Xiaoyang Wu, Huanxin Zhang, Jun Chen, Shuai Shang, Jiakuo Yan, Yao Chen, Xuexi Tang, Honghai Zhang

**Affiliations:** 1 College of Life Science, Qufu Normal University, Qufu, Shandong P.R. China; 2 College of Marine Life Sciences, Ocean University of China, Qingdao, P.R. China

## Abstract

Next Generation Sequencing has been widely used to characterize the prevalence of fecal bacteria in many different species. In this study, we attempted to employ a low-cost and high-throughput sequencing model to discern information pertaining to the wolf microbiota. It is hoped that this model will allow researchers to elucidate potential protective factors in relation to endangered wolf species. We propose three high-throughput sequencing models to reveal information pertaining to the micro-ecology of the wolf. Our analyses advised that, among the three models, more than 100,000 sequences are more appropriate to retrieve the communities’ richness and diversity of micro-ecology. In addition, the top five wolf microbiome OTUs (99%) were members of the following five phyla: Bacteroidetes, Fusobacteria, Firmicutes, Proteobacteria, and Actinobacteria. While *Alloprevotella, Clostridium_sensu_stricto_1, Anaerobiospirillum, Faecalibactreium* and *Streptococcus* were shared by all samples, their relative abundances were differentially represented between domestic dogs and other wolves. Our findings suggest that altitude, human interference, age, and climate all contribute towards the micro-ecology of the wolf. Specifically, we observed that genera *Succinivibrio* and *Turicibacter* are significantly related to altitude and human interference (including hunting practices).

## Introduction

A large number of bacterial species are known to colonize various anatomical sites within the body^1–3^. For instance, the colon is estimated to contain between 10^10^ and 10^14^ bacterial cells^4^. These extremely large microbial populations display significant diversity and have evolved novel mechanisms that facilitate their proliferation and maintenance^5^. The associated mechanisms have been the focus of a large number of studies worldwide^6^. Many studies have demonstrated that microbiota play important roles in the biogeochemical cycling of carbon, nitrogen, and phosphorus. Furthermore, these microbiological communities are capable of facilitating the decomposition of organic material and the extraction of nutrients from the resultant matter.

The focus of this research involved the microbiota of the native wolf with a particular emphasis on microbial diversity. It has been reported that microbial diversity plays an important role in the maintenance of host health^7^. It has also been suggested that factors such as age and gender affect the composition of microbial populations^8^. It is likely that mutualism between -specific characteristics, microbial richness, and diversity is an important phenomenon in host health^9^.

The traditional method that has been used to study microbial diversity in animals involves morphological and biochemical examination of colonizing microorganisms following *in vitro* isolation and purification techniques^10^. However, this method has many inherent drawbacks and limitations. For instance the vast majority of fecal microbes cannot be cultured. Furthermore, only 0.1%–10% of microbial species can be differentiated using these methodologies^11^. Another method that can be used to analyze microbial diversity depends on traditional molecular biology techniques, including denaturing gradient gel electrophoresis (DGGE)^12^, temperature gradient gel electrophoresis (TGGE)^13^, restriction fragment length polymorphism (RFLP) analysis^14^, and terminal restriction fragment length polymorphism (T-RFLP) analysis^15^. These methods do not exhibit many of the drawbacks associated with morphological and biochemical methods. Each of these approaches has contributed to our understanding of the importance of microbial diversity in hosts; however, all of these approaches have limitations in relation to both the quantity and quality of the data generated.

In recent years, a cross-disciplinary approach that has utilized both bioinformatics and molecular biological techniques is being adopted to study microbial diversity with dedicated academic programs featuring utilization of associated techniques. DNA sequencing technologies and applications have evolved extremely rapidly and the associated platforms currently facilitate the assimilation of big genome data, which are crucial for many research areas and applications^16^. From Sanger sequencing technologies in the mid-twentieth century to the utilization of High-Throughput sequencing platforms at the beginning of the twenty-first century, sequencing analyses have helped to develop our understanding of fecal bacterial profiles. Indeed, Sanger sequencing technologies have helped scientists to overcome several of the limitations pertaining to more traditional approaches that permit bacterial identification^17^. Furthermore, high-throughput sequencing technologies have provided quicker turnaround times and increased sequence data assembly rates. The latter technologies also facilitate low-cost utilization, higher coverage rates, higher accuracy, increasing read-lengths, and paired-end sequencing^18^. Thus, Next Generation Sequencing (NGS) platforms are now more widely applied in the analysis of fecal bacteria^19–22^. These analyses favor low-cost, high-throughput methodologies; however, the associated cost and throughput is somewhat dependent on the application requirements. In this article, we analyzed a substantial body of High-Throughput sequencing data to determine an appropriate balance that meets high-throughput and low-cost requirements.

Previous studies have demonstrated the existence of microbiota diversity and richness in animals including the panda and the monkey^23–28^. These studies investigated the latter parameters in both species and subspecies^29, 30^. Several studies pertaining to both the wolf and the dog have also been conducted in this field. Zhang *et al*. reported data relating to microbiota diversity and richness in the wolf following an analysis that facilitated the cloning of bacterial 16S rRNA gene amplicons^31^. Suchodolski *et al*. showed microbiota diversity in the intestinal segments of dogs^32, 33^. Additional studies reported data pertaining to microbiomes of ill-conditioned fecal^32, 34, 35^. However, the methods that were used in these analyses and the associated data sizes that were generated are outdated. Furthermore, there is no reference to the dog microbiome in these wolf-specific studies. The dog is a subspecies of the wolf; thus, to attain a more generic understanding of microbiota diversity and richness in related subspecies, samples from both the dog and the wolf should be studied together.

As part of this study, eighteen dogs and wolves were selected to characterize microbiota composition using multi-group sequencing analysis. The wolf is now listed on the International Union for Conservation of Nature and Natural Resources endangered species list of threatened species^36^. Over the last number of centuries, the wolf has been one of the most widely distributed animal species in the world^31, 37^. However, the population of this species has rapidly declined and is currently threatened by habitat loss and hunting practices. For all this, scientists will continue to work hard to explore the protection mechanism^38^. Wolves are independent and are able to adapt to the environment. However, its independent and adaptation mechanisms are unknown. At present only semi-artificial, semi-wild, and bowel disease is a significant aspect of health. All of this needs to be solved. Intestinal microbes are part of a complex ecosystem. They have a mutual relationship with the host and play an essential role in maintaining the host’s health. Following a comprehensive analysis of multi-group sequencing data, this report reveals the composition of the wolf fecal microbiota while also describing factors that influence this composition. This study will provide valuable basic data that should help in efforts related to the future conservation of wolves.

## Results

### OTUs and Taxonomic composition of the fecal microbiomes

**Figure 1:**
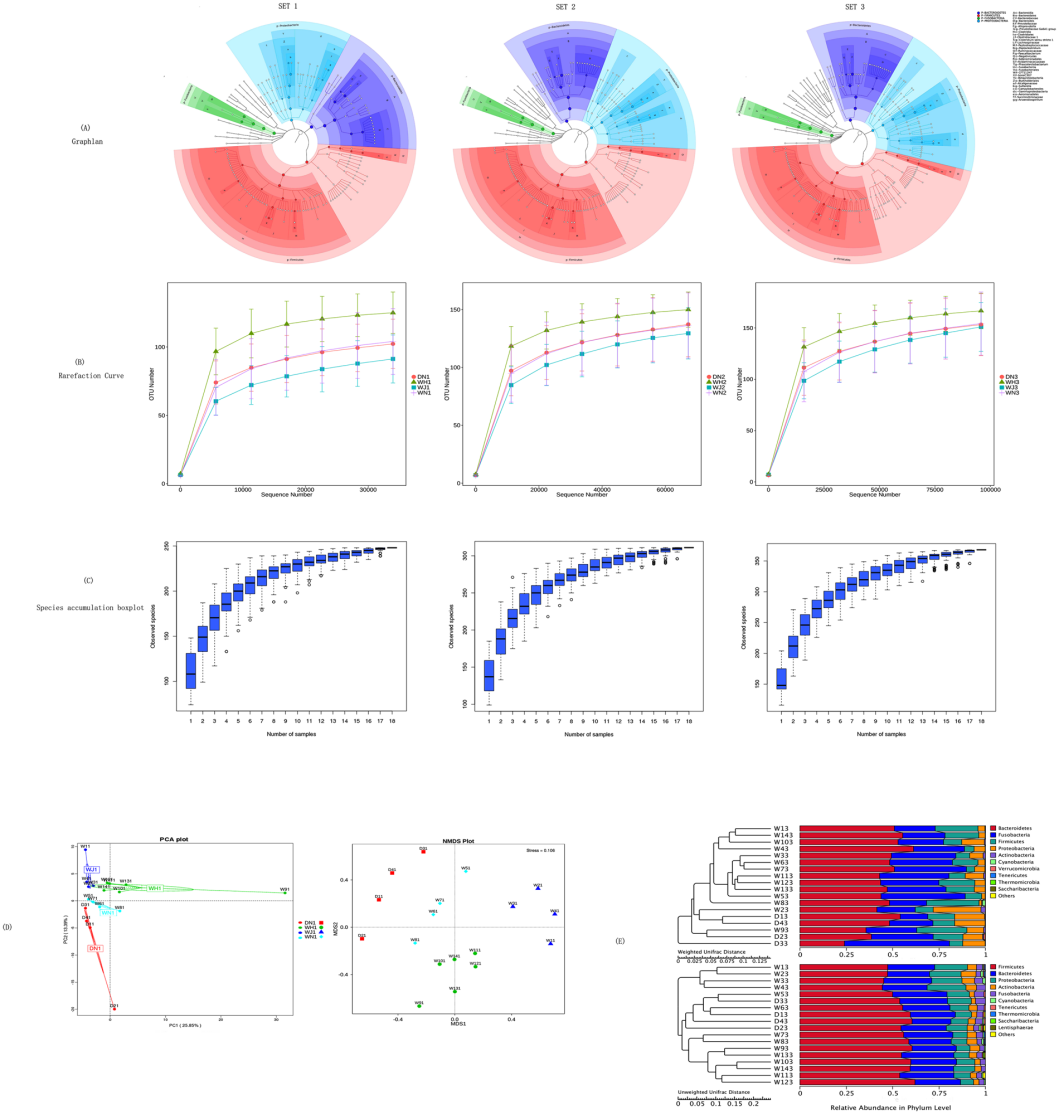
(**A**) The circle from inside to outside in turn was on behalf of different classification levels, and species abundance is proportional to the size of the circle, the different colors represent different phyla, and abundance of the top 40 species was showed in solid circle; (**B**) Rarefaction Curve: rarefaction analysis of V3-V4 16S data from three wolf data sets. There are three data set in the figure (SET1, SET2, SET3) and each line represents a group. Analysis was performed on a random 33952-, 67261-, 95787 sequence subset from each data sets. (**C**) Species accumulation boxplot: the arisen speed of new species with increasing samples; (**D**) Principal coordinate analysis plots of unweighted UniFrac metrics for wolf miocrobiotas. Each dot represents individual microbiota samples obtained from DN, WH, WJ, WN; the figure on the left is a jackknifed clustering of the wolf in the weighted uniFrac dataset and unweighted dataset. The figure on the right is phylum assignment of V3-V4 16S sequences from wolves.

From the three data sets, we obtained >50000, >100000 and >150000 raw sequences, across a total of 18 samples, respectively (Table 1). To avoid analytical variation, identical protocols, including the mothur MiSeq-SOP, were utilized. Following denoising steps, effective and unique sequences were obtained. The average numbers of unique sequences were 1916, 3339, and 4977 (Table 1). Clustering was performed using Uparse, and a total of 118, 151 and 170 OTUs were generated for the three data sets, respectively (Table 1). To explore the dominant bacterial species, the results were annotated using GraPhlAn (Fig. 1A)^39^.

**Table 1:** Comparison of phylotype coverage and diversity estimation of the 16S rRNA gene libraries at 3% dissimilarity from the analysis of the basic statistics information.

	Raw	Effect	Unique	OTU	Shannon	Simpson	Chao 1	ACE
SET 1	51450	49534	1916	118	3.8	0.85	120.56	118.67
SET 2	102898	99559	3339	151	4.1	0.90	154.93	154.93
SET 3	150196	141192	4977	170	4.1	0.90	176.45	176.21

Associated histograms were generated with species annotation to six classification levels. Twenty phyla were observed following this analysis. The five most prominent OTUs (99%) were from the following five phyla; Bacteroidetes, Fusobacteria, Firmicutes, Proteobacteria, and Actinobacteria. This observation was consistent among the three data sets (Fig. 1E). Upon comparison of the associated phyla, the relative abundance of Bacteroidetes was as follows: WJ > WN > WH > DN; the relative abundance of Fusobacteria was as follows: WN > DN > WH > WJ; the relative abundance of Firmicutes was as follows: WH > WN > DN > WJ; the relative abundance of Proteobacteria was as follows: DN > WJ > WH > WN; and the relative abundance of Actinobacteria was as follows: WJ > WH > DN > WN. Upon analysis of climate-mediated effects in relation to microbiota composition, we observed positive and negative correlations with respect to climate for Bacteroidetes and Firmicutes, respectively. Proteobacteria and Actinobacteria were observed to positively correlate with wolves exposed to human interference practices, while Fusobacteria were observed to be negatively correlated with these interference effects. Interestingly, Cyanobacteria were only observed in the WN, and Verrucomicrobia were observed exclusively in the DN. At the genus level, the most frequently detected genera were *Bacteroides, Alloprevotella, Sutterella, Clostridium_sensu_stricto_1, Anaerobiospirillum, Prevotellaceae_Ga6A1_group, Helicobacter, Faecalibacterium, Phascolarctobacterium*, and *Lachnoclostridium*. In addition, *Clostridium_sensu_stricto_1* was most prevalent in the WN group and *Phascolarctobacterium* and *Lachnoclostridium* were the most prevalent genera in the WH group.

### Comparison of microbial group diversity

Following alpha diversity analysis, the indices for bacterial richness and the diversity of OTUs at a 3% sequence dissimilarity level are summarized in Table 1. Increased community richness was observed following the analysis of increased amounts of data (Table 1). The richness index values for SET 2 and SET 3 were almost equal and were greater than the associated value for SET 1 (Table 1). In SET 3, there was no significant difference in community richness between the different samples; however, the Shannon and Simpson indices for wolves in Inner Mongolia were reduced compared with other areas (Table 1). Following analysis of the rarefaction curve (Fig. 1B) and species accumulation boxplot (Fig. 1C) diagram, it is noticeable that the curves are on a trajectory towards a constant equilibrium.

**Figure 2:**
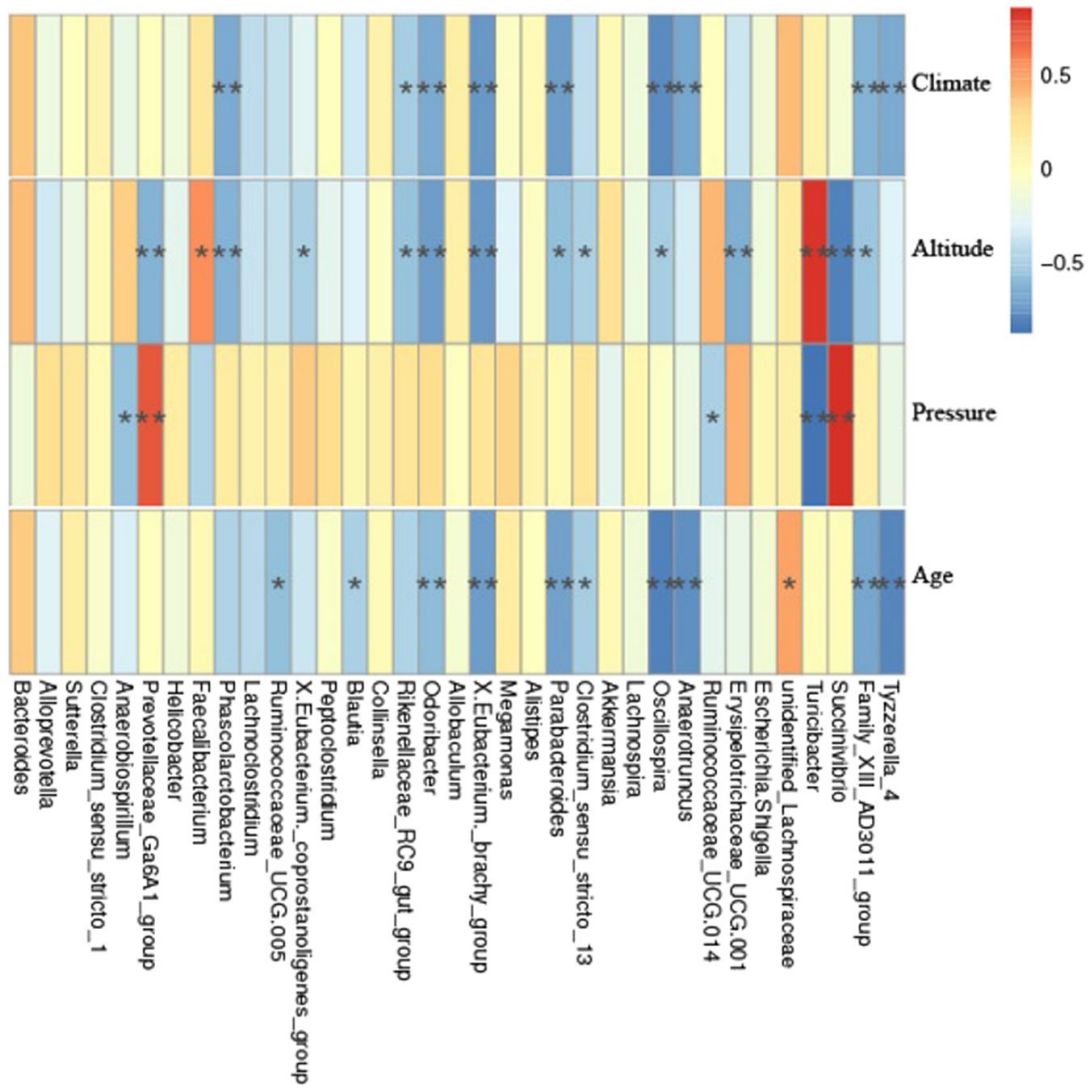
The ordinate is the environment information and the abscissa is species. The corresponding values of heatmap are spearman rank relational coefficient. The * means significant (P < 0.05) and the ** means remarkable significant (P < 0.01).

We are told from two model OTU diagrams (linear model PCA and nonlinear model NMDS) (Fig. 1D) that the samples from one groups are collected together, so the difference and consistency were showed phenomenally. In order to elucidate further the cluster structures for wolves, we attempted to map UPGMA hierarchical clustering for SET 3 (Fig. 1E). However, the cluster analysis generated following Weighted Unifrac and Unweighted Unifrac analyses was more complex (Fig. 1E). The heatmap from the Spearman analysis suggested that *Succinvibrio* richness negatively correlated with altitude and positively correlated with pressures (Fig. 2). Conversely, *Turicibacter* richness positively correlated with altitude and negatively correlated with human interference (Fig. 2). We also observed that the prevalence of some bacterial phyla correlated both negatively and positively with age. Additional details relating to observed correlations are presented in Fig. 2.

### Optimization selection of the three models

**Figure 3:**
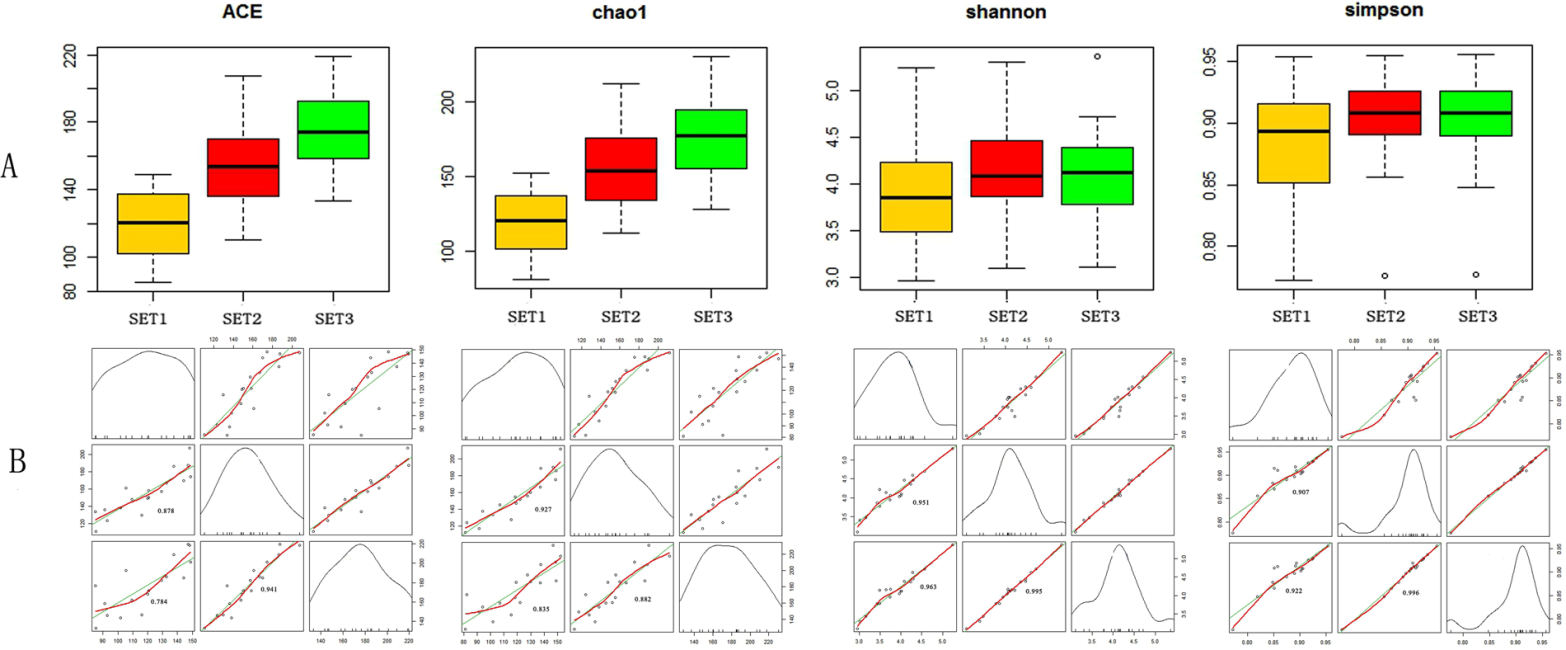
(**A**): Boxplot with whiskers from minimum to maximum; (**B**): scatter diagram and Goodness of Fit.

Utilizing identical protocols, we get more diversity index (ace, chao1, simpson, shonnon) from three models. We realize of visualizing processing to the basic statistics data, such as minimum, maximum, median, averages and so on (Fig. 3A). From Fig. 3A, the community richness (ace, chao1) increased with the data increases. However, the community diversity is different from the patterns of community richness. The community diversity of SET 1 has significantly different from the SET2 and SET3. Among them, the median, average and numeric range is similar in SET2 and SET3 and is higher than the SET1. In other words, the development of SET2 and SET3 has a similar stable trend. In particular, the SET3 can reflect the outliers. In the Goodness of fit on three data set of analysis, we added the scatter plot and regression curve. Our normalization shows the probability distributions. The data of SET 1 is decentralized but the data of SET 2 and SET 3 were more centrally. Moreover, the data of SET 2 and SET 3 were almost unanimously and tend to be stable. On the other hand, SET 2 and SET 3 reflect the community diversity is largely consistent (Shannon: R^2^ = 99.5%, Simpson: R^2^ = 99.6%) (Fig. 3B).

### Comparison of the gut microbiota of domestic dog and wolf from the SET 3

**Figure 4:**
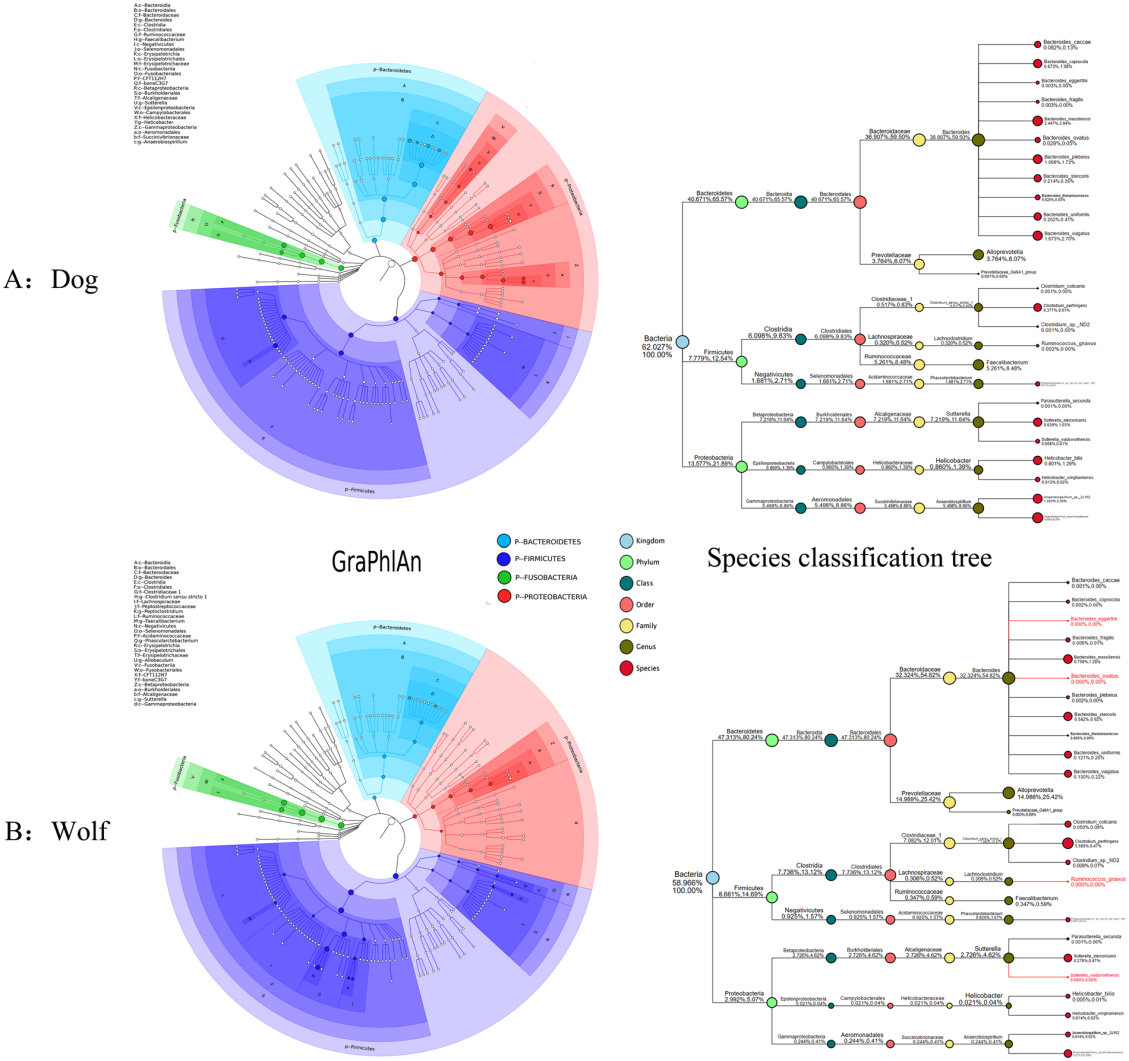
GraPhlAn visualization of annotated phylogenies and taxonomies. We comprise of microbial community abundances between dog and wolf using the phylogenetic tree on all available microbiota. Colors and background annotation highlight bacterial phyla.

**Figure 5:**
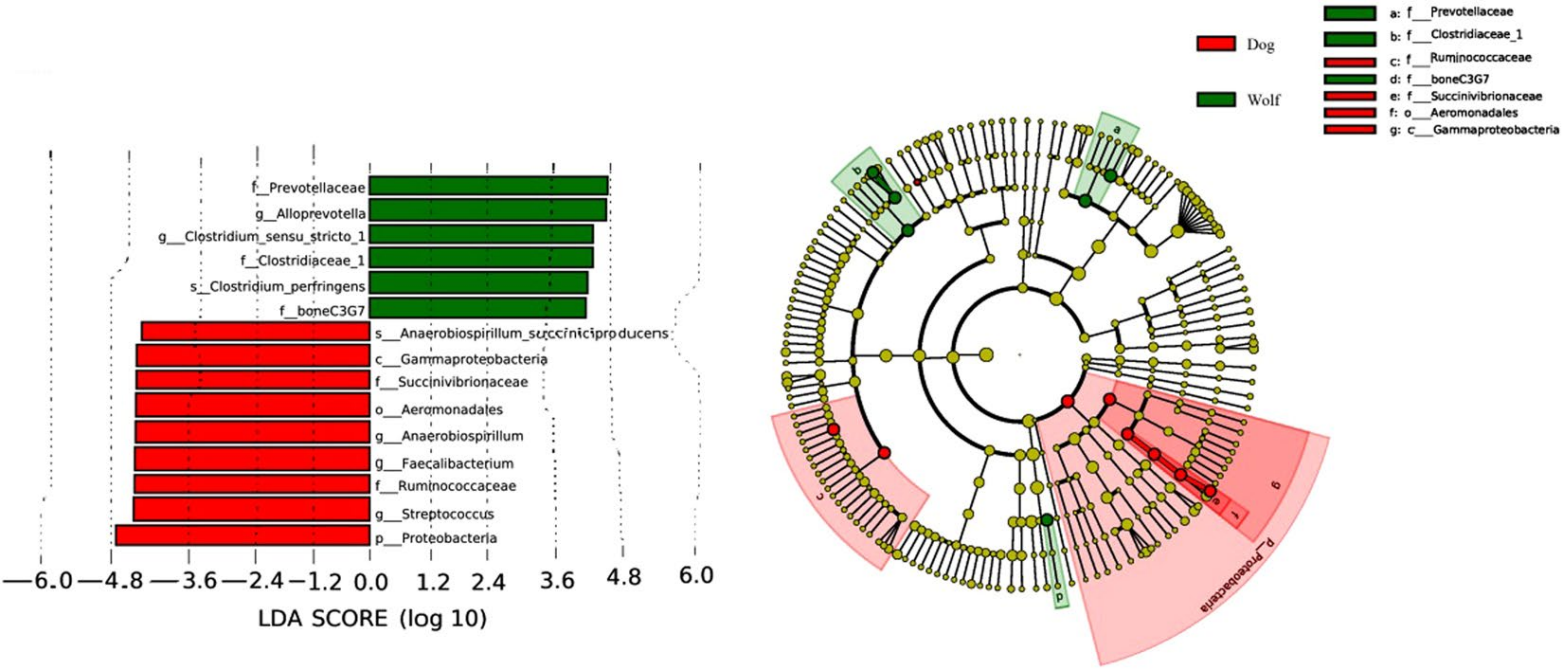
Bacteria differentially represented between the domestic dogs and the wolves identified by linear discriminant analysis coupled with effect size (LEfSe). Histogram showing Bacteria that are more abundant in the domestic dogs (red color) or the wolves (green color) ranked by linear discriminant analysis (LDA) score and phylogenetic tree also showing the biomarker with significant difference.

We characterized the gut microbiota of 14 wolves and 4 domestic dogs. The wolves’ number of OTUs is 167 and the domestic dogs’ number of OTUs is 170. Five phyla including Bacteroidetes, Fusobacteria, Firmicutes, Proteobacteria, Actinobacteria were predominant bacterial. In addition, Cyanobacteria (0.1391%) was only detected in wolf and Verrucomicrobia (0.2312%) only detected in domestic dog. Particular phyla were at low relative abundances. We also use GraPhlAn (Graphical Phylogenetic Analysis), a computational tool that produces high-quality, compact visualizations of microbial metagenomes(Fig. 4)^39^. Exploiting the shared and unique bacterial taxa between the gut microbiota of the domestic dog and the wolf is also our aim. It was unexpected that *Cyanobacteria* exist only in the wolf and *Verrucomicrobia* exists only in the domestic dog. We used linear discriminant analysis effect size (LEfSe) to identify genus differentially represented between the wolf and the domestic dog. While *Alloprevotella, Clostridium_sensu_stricto_1, Anaerobiospirillum, Faecalibactreium* and *Streptococcus* were shared by all samples, their relative abundances were differentially represented between the two (Fig. 5). The genus *Alloprevotella* and *Clostridium_sensu_stricto_1* is significantly higher in the wolf than in the domestic dog. In contrast, *Anaerobiospirillum, Faecalibactreium* and *Streptococcus* is more abundant in the domestic dog (Fig. 5). *Streptococcus* only exists in the domestic dog and was absent from the wolf. Whether or not the gut microbiota is involved within digestion needs further investigation.

## Discussion

The objective of this study was to determine an appropriate balance between sequence data generation and cost in relation to an analytical model that could help to protect the valuable wildlife-wolf. The study explored the composition of microbiota of wolves following high-throughput sequencing analysis.

With the rapid development of Next Generation Sequencing (NGS) technology, there is an onus on micro-ecologists to use high-throughput sequencing platforms to help solve complex biological problems. However, high-throughput sequencing can be relatively costly. Nevertheless, high-throughput sequencing generates accurate results that facilitate complex sequence analysis. Upon analysis of these results, we advised that, >100,000 sequences can restore the information pertaining to community richness and micro-ecological diversity.

Traditional paradigms suggest that the generation of greater amounts of sequencing data will facilitate more accurate results. Thus, we chose the SET3 data to explore the microbiota of wolves. Previous work in this field performed using phylogenetic analysis of 16S rRNA gene sequences revealed distal fecal bacterial diversity in wild wolves and dogs^40–42^. The latter studies on bacterial diversity were predominantly based on cloning bacterial 16S rRNA gene amplicons. This method was deemed the most advanced for these analysis types; however, there are limitations associated with this form of analysis including limitations relating to the amount of data generated. Moreover, the gray wolf (*Canis lupus*) encompasses both wolf (*Canis lupus linnaeus*) and dog (*Canis lupus familiaris*) species. Unfortunately, these studies only revealed information regarding singular dog or wolf species. Therefore, we attempted to revisit this area of research using Next Generation Sequencing (NGS) technology and the Illumina MiSeq (Illumina MiSeq, USA) platform. Our results demonstrated that Bacteroidetes (46.48%), Fusobacteria (30.54%), Firmicutes (13.46%), Proteobacteria (8.83%), Actinobacteria (0.53%), Cyanobacteria (0.10%), Verrucomicrobia (0.01%), Tenericutes (<0.01%), Saccharibacteria (<0.01%) and Lentisphaerae (<0.01%) were the most prevalent phyla in wolf microbiota. However, Chen *et al*. identified the presence of five phyla (Firmicutes (60%), Bacteroidetes (16.9%), Proteobacteria (9.2%), Fusobacteria (9.2%) and Actinobacteria (4.6%)) and other reports only detected seven bacterial phyla including the additional phyla Spirochaetes and Tenericutes^33, 34, 41, 43, 44^.

Upon analysis of different variables, we observed that microbiota composition in wolves is dependent upon a number of factors including age, altitude, pressure, and climate. Another interesting observation included the fact that one of the groups, DN, exhibited several noticeable differences from the other groups. This occurrence is most likely because the associated group consisted of dogs, which are a subspecies of wolf. Following on from our analysis and contributions from other studies pertaining to affecting factors^2, 3, 7, 45–57^, it is apparent that the micro-ecological environment of wolves is responsible for defense against unfavorable environmental factors. For instance, *Succinivibrio*, *Turicibacter*, and *Prevotellaceae_Ga6A1_group* appear to be involved in protection against pressures associated with human interference of wolf species (reference Spearman analysis diagram, Fig. 2).

In order to investigate the relationship between bacterial populations and environmental factors, we performed a literature search focusing on specific bacterial genera. The Spearman’s rank correlation for the researched bacterial genera was greater than 0.7 and was extremely significant (P < 0.01). *Succinivibrio* and *Turicibacter* were observed to correlate with altitude and human interference. Members of the genus *Succinivibrio* require carbon dioxide for growth. These bacteria are anaerobic and ferment organic matter produced by the Krebs cycle to generate acetic acid and succinic acid. *Turicibacter* species are facultative. In addition, they produce acid but not gas. Thus, it is likely that a greater abundance of *Succinivibrio* and *Turicibacter* can facilitate Krebs Cycle progression, thereby resulting in greater absorption of carbon dioxide. Therefore, as environmental pressure increases, fecal bacteria can help to reduce the amount of energy produced, thereby maintaining homeostasis in the body. These findings relating to the prevalence of *Succinivibrio* and *Turicibacter* in the microbiota of wolves may help to explain how this energy conservation manifests itself under adverse conditions.

Gut microbiota of wolf is negatively associated with the pressure from humans while domestic dogs are just the opposite. The wolves are not interested in human social cues and will be a threat. Conversely, the domestic dogs care about the human social cues. After thirty-thousand years of domesticating, the dog has gradually understood and adapted to humans while the wolf continued in a state of enmity. In order to satisfy the different relations, the organisms may make some changes, which include the gut microbial.

In summary, the three data set determined an appropriate balance between sequence data generation and cost for the primary scientific research workers. The findings obtained in our study also provided a special insight into the ecology and biodiversity of the wolf gut microbiome. We observed that the microbiota composition was dependent on habitat and four different analyzed factors were important in determining the prevalence of microbiological genera in the wolf fecal. These microbial communities co-exist with their host and play important roles in the long-term evolution of the host^3^. These findings provided a powerful tool for characterization of the micro-ecological environment of threatened wildlife species, thereby allowing us to identify factors that might be important in population maintenance and protection.

Our samples in this study were taken from several representative geographical areas in China. We all know that wolves are globally distributed and inhabited in different ecological environment. Further research is necessary to test our findings in more wolves, as well as to better understand the trade-off between nutrition and health via shifts in gut microbiota composition. The ultimate goal is that all wild animals around us can survive and reproduce better.

## Methods

### Sample collection

Fecal samples were collected from wolves in China. They were all raised semi-freely in the Care Centre with raw meat and water before the fecal samples were collected. None of the wolves received any treatment (e.g., antibiotic therapy) that would be expected to have an impact on the composition of the intestinal microbial community. All procedures that were performed on animals were conducted in accordance with the ethical standards of the Qufu Normal University Animal Care and Use Committee (Permit Number: QFNU2015-002). None of the animals were harmed during the collection of fecal samples. Most scientists divide the wolves in China into five subspecies and forms: *Canis lupus desertorum Bogdanow*, *C. I. filchneri* Matschie, *C. I. chanco* Gray, Inner Mongolia (eastern part), *C. I. Nei-Mongol* form (western and mid part) and *C. I. South* China form. In the economically developed eastern China, the human disturbance is very powerful. The result is that the wolves’ population has declined drastically. Especially in the southern provinces, it is not clear whether wolves recorded in the southern provinces represent permanent populations, or a steady stream of individuals migrating from the northern provinces^58–60^. However, the extreme conditions would have a serious impact on body function and gut microbiome. So, our study collected three subspecies and forms: *C. I. chanco* Gray (WH), *C. I. Nei-Mongol* form (WN and DN) and *C. I. South* China form (WJ). These environments are less extreme. Details pertaining to the animals chosen are presented in Table 2. Different species-specific territories exhibit different climates and the three regions chosen as part of this analysis represented the different climates in China. In China, rainfall is one of the most important climate-mediated effects and we used rainfall as an indicator of climate. From previous studies, we hypothesized that human interference affects nervous system and sample microbiome composition as well^61–64^. Thus, pressures exerted by human interference were investigated for their effects on the micro-ecology of the dog and the wolf. The fecal matter of one sample is collected in triplicate in three days. Fecal collections were immediately made after defecation during the early morning. And fecal samples were immediately placed into sterile plastic tubes frozen in sample containers and carried to the lab and stored at −80 °C until DNA extraction.

**Table 2:** Details of the animal information.

	Age	local	SET1	SET2	SET3	Altitude (m)	Climate-value (mm)	Pressure-value
**Wolf**								
WJ	4	Jiangxi	W11	W12	W13	200	2015	3
	12	Jiangxi	W21	W22	W23	200	2015	3
	5	Jiangxi	W31	W32	W33	200	2015	3
	12	Jiangxi	W41	W42	W43	200	2015	3
WN	4	Inner Mongolia	W51	W52	W53	1000	328	1
	4	Inner Mongolia	W61	W62	W63	1000	328	1
	2	Inner Mongolia	W71	W72	W73	1000	328	1
	1	Inner Mongolia	W81	W82	W83	1000	328	1
WH	1.5	Henan	W91	W92	W93	75	145	2
	1.5	Henan	W101	W102	W103	75	145	2
	1.5	Henan	W111	W112	W113	75	145	2
	1.5	Henan	W121	W122	W123	75	145	2
	1.5	Henan	W131	W132	W133	75	145	2
	1.5	Henan	W141	W142	W143	75	145	2
**Dog**								
DN		Inner Mongolia	D11	D12	D13	1000	328	1
		Inner Mongolia	D21	D22	D23	1000	328	1
		Inner Mongolia	D31	D32	D33	1000	328	1
		Inner Mongolia	D41	D42	D43	1000	328	1

### DNA extraction, PCR amplification, and 16S rRNA sequencing

Total community genomic DNA was extracted from all fecal samples using a QIAamp DNA Stool Mini Kit (Qiagen, Germany) as per the manufacturer’s instructions. The DNA was amplified using specific primers that targeted the V3-V4 region of the 16S rRNA bacterial gene. The primers also carried the Illumian MiSeq sequencing adapter (16S Amplicon PCR Forward Primer: CTACGGGNGGCWGCAG and 16S Amplicon PCR Reverse Primer: GACTACHVGGGTATCTAATCC)^7^. The PCR mix was prepared using the KAPA HiFi Hot Start Ready Mix (2×) (TaKaRa Bio Inc., Japan). PCR conditions for MiSeq are described in Wu *et al*. (2016). Amplicons of 16S rDNA were purified using AMPure XP beads (Beckman, USA). The final DNA samples that were extracted from the fecal samples were pooled in equal concentrations prior to sequencing on the Illumina MiSeq platform (Illumina MiSeq sequencing system, USA) in our laboratory.

### Data selection and arrangement

Greater than 150,000 reads per sample were generated using the Illumina MiSeq platform. In order to assess an optimal balance between throughput and cost, we varied the numbers of reads per sample. The variations, which were randomly selected, included 50,000 reads per sample, 100,000 reads per sample, and 150,000 reads per sample, respectively. During sequencing, single DNA molecules are randomly bound to the surface of the flow cell and bridge-amplified to form clusters. Reads in the fastq file are subsequently randomly generated. The head command line subsequently permits selection of the number of sequences using the Linux operation system. The three data sets represent three different data sizes (30%, 60%, and 100%). The different sequencing data sizes represent differing sequencing depths. Researchers in the area of intestinal micro-ecology predominantly use between 20,000 and 100,000 sequencing reads^3, 7, 16, 31, 51, 65^. Thus, our three data sets expand the upper range and encompass much of the conventional range of use.

### OTUs and fecal bacteria

First, operational taxonomical units (OTUs) were analyzed for each sample with a 97% sequence similarity cutoff value. Secondly, a summary of all taxonomic information was generated using RDP Classifier version 2.2^66^. The phylogenetic relationship was elucidated using GraPhlAn. Finally, to standardize results, the lowest number of sequences from each sample was randomly selected and different data sets were observed to contain different homogeneous sequences.

### Diversity analysis

Alpha diversity analysis facilitated the construction of a rarefaction curve and species accumulation boxplot. These were used to describe the number of OTUs and species as a function of sampling effort^7^. Next, richness of the associated communities was compared based on the ACE estimator and the Chao1 estimator. Community diversity was subsequently analyzed using both the Simpson index and the Shannon index.

Beta diversity analysis was used to determine microbiota composition diversity between the individuals using the linear Principal Component Analysis (PCA) model and the nonlinear Non-Metric Multi-Dimensional Scaling (NMDS) model. Wolf microbiota phylogenetic analysis was performed using an Unweighted Pair-group Method with Arithmetic Mean (UPGMA) and the associated phylogenetic trees were based on the Weighted Unifrac and Unweighted Unifrac values.

To understand the correlation between parameters including attitude, pressure, climate and age, the Spearman correlation and the Mantel test correlation were calculated. LDA Effect Size (LEfSe) can search for a Metagenomic biomarker between the two groups and the biomarker is statistically significant (P < 0.05).

We analyzed the three data sets using the Mothur (Version 1.36.1)^67^, Qiime (Version 1.7.0)^68^, Uparse (Uparse v7.0.1001)^69^, PyNAST (Version 1.2)^70^ and the following disgrams were made by R (R version 3.3.1)^71^.

### Statistical analysis of three models

For statistical analysis of the three models, we use identical protocols to calculate four kinds of diversity indices. Using the boxplot, we performed the preliminary observation on some basic statistics. The goodness of fit describes how well it fits a set of observations. A scatter plot can suggest various kinds of correlations between variables and analyzing join level for regression model. At last, we make an optimization selection. All analysis was carried out using R (R version 3.3.1).

### Availability of data and materials

We upload our raw sequences about this research on the Sequence Read Archive (accession number SRP089855).
